# Risk factors for surgical site infections after hysterectomy: A systematic review and meta-analysis

**DOI:** 10.12669/pjms.42.3.13718

**Published:** 2026-03

**Authors:** Yaqing Yue, Tiantian Zhao

**Affiliations:** 1 Yaqing Yue, Department of Gynecology, Weifang People’s Hospital, Weifang, Shandong Province 261000, P.R. China; 2 Tiantian Zhao, Department of Gynecology, Weifang People’s Hospital, Weifang, Shandong Province 261000, P.R. China

**Keywords:** Endometrial Cancer, Hysterectomy, Meta-analysis, Risk Factors, Surgical Site Infections, Systematic Review

## Abstract

**Background & Objective:**

Surgical site infections (SSIs) are a common complication following hysterectomy that is associated with higher morbidity, prolonged hospitalization, and a healthcare burden. Identifying key risk factors for SSIs will allow the development of effective prevention measures. Our objective weas to review and analyze potential risk factors associated with SSIs after hysterectomy, focusing on modifiable and non-modifiable contributors.

**Methodology:**

PubMed, EMBASE, Scopus, and CENTRAL databases were searched based on the PRISMA guidelines. The last electronic search was carried out on 30^th^ January 2025 to include studies to date. Sixteen studies with 165,589 participants were included. Random-effects meta-analysis was done to calculate odds ratios (OR) and 95% confidence intervals (CI). Heterogeneity was assessed using the I² statistic. *P* < 0.05 was considered significant. The protocol was registered in PROSPERO (CRD42025629931).

**Results:**

A total of 16 studies were included in this review. Key risk factors for SSIs included obesity [Body Mass Index (BMI) >30 kg/m²; OR = 3.34, 95% CI: 2.45–4.56], diabetes mellitus (OR = 1.82, 95% CI: 1.41–2.35), smoking (OR = 1.76, 95% CI: 1.37–2.26), and prolonged operative time (>2 hours; OR = 1.59, 95% CI: 1.19–2.11). Procedural factors such as open surgical approaches (OR = 2.88, 95% CI: 2.24–3.72) significantly increased SSIs risk. Additional contributing factors included prolonged hospital stays (>1 day; OR = 2.47, 95% CI: 1.78–3.43) and bowel resection (OR = 3.07, 95% CI: 1.71–5.49).

**Conclusion:**

Obesity, diabetes, smoking, and prolonged operative time are risk factors for SSIs that may be considered as modifiable for intervention. Evaluating these risk factors in patients undergoing hysterectomy may significantly reduce the burden of SSIs, improve outcomes, and lower healthcare costs.

**Registration No.** (PROSPERO: # CRD42025629931).

## INTRODUCTION

Endometrial cancer (EC) and Cervical cancer (CC) are significant global health concerns and are among the most common gynecological malignancies leading to mortality among women.^1^ Just in 2020, 417,367 new cases of EC were reported worldwide, with more than 97,370 deaths attributed to the disease, accounting for nearly 4% of all cancer-related deaths in women annually.^2^ Hysterectomy plays an essential role, including both benign conditions—such as uterine fibroids, abnormal uterine bleeding, and endometriosis—as well as malignant conditions like EC and CC, offering both curative and palliative outcomes.^3^ However, this approach is associated with a risk of postoperative complications, such as surgical site infections (SSIs) that contribute to substantial morbidity, impact patient recovery, and overall treatment efficacy,^4^,^5^ affect quality of life, and place a financial strain on healthcare systems.^6^,^7^ Therefore, understanding and mitigating the risk factors for SSIs in patients undergoing hysterectomy is essential to improving surgical outcomes and reducing healthcare costs.

Numerous studies have explored the potential risk factors associated with SSIs after hysterectomy.^8^ Commonly identified predictors include high body mass index, diabetes mellitus, surgical procedure and operative time, and the American Society of Anesthesiologists (ASA) score. These factors, often interrelated, highlight the complex interplay of patient characteristics, surgical techniques, and perioperative management in determining the risk of infection. Despite these insights, there is still a significant variability in the findings across different studies. Inconsistencies in study designs, populations, and definitions of SSIs have contributed to a lack of consensus regarding the relative importance of specific risk factors.

As SSIs are associated with a substantial healthcare burden, a comprehensive evaluation of the risk factors associated with this complication in the context of a hysterectomy is necessary. Identifying modifiable and non-modifiable factors could guide clinicians in implementing targeted preventive strategies, optimizing perioperative care, and ultimately improving patient outcomes. This meta-analysis aims to assess the link between key risk factors and the occurrence of SSIs after hysterectomy.

## METHODOLOGY

This systematic review and meta-analysis were done based on the Preferred Reporting Items for Systematic Reviews and Meta-Analyses (PRISMA) guidelines,^9^ and pre-registered with the International Prospective Register of Systematic Reviews (PROSPERO: # CRD42025629931).

### Research question:

The research question was formulated using the PICO framework to address the factors contributing to SSIs after a hysterectomy. Specifically:

### Population:

Women undergoing hysterectomy.

Intervention/Exposure: Potential risk factors such as obesity, diabetes, surgical approach, operative time, and ASA score.

### Comparison:

Patients without these risk factors or with lower levels of exposure.

### Outcome:

Incidence of SSIs.

The primary research question was: What are the risk factors associated with SSIs after hysterectomy?

### Search strategy:

A comprehensive search of PubMed, Embase, Cochrane Library (CENTRAL), and Scopus databases was done using MeSH key words. The key words used for developing the search strategy include Hysterectomy”; “Uterus Removal”; “Uterine Surgery”; “Surgical Wound Infection”; “Surgical Site Infection”; “Postoperative Infection”; “Wound Infection”; “SSI”; “Risk Factors”; “Risk Factor”; “Associated Factors”; “Predictors”; “Determinants”. No restrictions were placed on the year of publication and language, to capture all potentially relevant data, but only studies published in English were considered. In addition to database searches, grey literature, conference abstracts, and the bibliography of included studies were searched to ensure comprehensiveness. The grey literature search was carried out using *www.opengrey.com*. The last electronic search was carried out on 30^th^ January 2025. The search strategy for all databases is provided in Table-I.

**Table-I:** Search strategy for digital databases.

Data	Details	Number
PubMED	("Hysterectomy"[Mesh] OR "Hysterectomy"[Title/Abstract] OR "Uterus Removal"[Title/Abstract] OR "Uterine Surgery"[Title/Abstract]) AND ("Surgical Wound Infection"[Mesh] OR "Surgical Site Infection"[Title/Abstract] OR "Postoperative Infection"[Title/Abstract] OR "Wound Infection"[Title/Abstract] OR "SSI"[Title/Abstract]) AND ("Risk Factors"[Mesh] OR "Risk Factor"[Title/Abstract] OR "Associated Factors"[Title/Abstract] OR "Predictors"[Title/Abstract] OR "Determinants"[Title/Abstract])	155
Scopus	(TITLE-ABS-KEY(hysterectomy OR "uterus removal" OR "uterine surgery")) AND (TITLE-ABS-KEY("surgical site infection" OR "surgical wound infection" OR "postoperative infection" OR "wound infection" OR SSI)) AND (TITLE-ABS-KEY("risk factor" OR "associated factor" OR "predictor" OR "determinant" OR "contributing factor"))	232
EMBASE	(’hysterectomy’/exp OR ’hysterectomy’ OR ’uterus removal’ OR ’uterine surgery’) AND (’surgical wound infection’/exp OR ’surgical site infection’ OR ’surgical wound infection’ OR ’postoperative infection’ OR ’wound infection’ OR ’SSI’) AND (’risk factor’/exp OR ’risk factor’ OR ’associated factor’ OR ’predictor’ OR ’determinant’ OR ’contributing factor’)	176
CENTRAL	([MeSH descriptor] "Hysterectomy" explode all trees OR hysterectomy OR "uterus removal" OR "uterine surgery") AND ([MeSH descriptor] "Surgical Wound Infection" explode all trees OR "surgical site infection" OR "surgical wound infection" OR "postoperative infection" OR "wound infection" OR SSI) AND ([MeSH descriptor] "Risk Factors" explode all trees OR "risk factor" OR "associated factor" OR "predictor" OR "determinant" OR "contributing factor")	149

### Study selection:

In the first stage of study selection, two reviewers (Y.Y, T.Z) independently screened titles and abstracts to assess relevance. Second, full texts of potentially suitable studies were evaluated using predefined eligibility criteria. Disagreements were resolved by discussion.

### Inclusion criteria:

- Studies reporting SSIs as an outcome after hysterectomy.
- Observational studies (cohort, case-control) and randomized controlled trials (RCTs).
- Studies reporting specific risk factors and their association with SSIs.
- Full-text articles available in English.

### Exclusion criteria:

- Studies without explicit reporting of SSIs.
- Case reports, reviews, or editorials.
- Studies without extractable data or overlapping datasets.

### Data Extraction:

Two reviewers (Y.Y, T.Z) independently used a standardized form in an Excel spreadsheet to retrieve study characteristics (e.g., author, year, design, sample size), definitions and incidence of SSIs, examined risk factors, and reported effect sizes and measures. Discrepancies were resolved by consensus, and missing data were sought by contacting study authors when feasible.

### Quality of studies or Risk of bias assessment:

Randomized Controlled Trials (RCTs) were assessed using the Cochrane Risk of Bias 2 (RoB 2) tool (https://methods.cochrane.org/bias/resources/rob-2-revised-cochrane-risk-bias-tool-randomized-trials), evaluating five domains: randomization process, intervention deviations, missing outcome data, measurement of outcomes, and selection of reported results. For observational studies, the Newcastle-Ottawa Scale (NOS) was applied to evaluate the selection of study groups, the comparability of groups, and the measurement of exposure or outcome (https://ohri.ca/en/who-we-are/core-facilities-and-platforms/ottawa-methods-centre/newcastle-ottawa-scale). Each study was assigned a quality score, and sensitivity analyses were done to examine the impact of including lower-quality studies.

### Data synthesis:

The extracted data were subjected to qualitative and quantitative analysis. A random-effects meta-analysis was conducted using RevMan 5.4.1v (Cochrane Collaboration, UK) to account for heterogeneity among studies. Effect sizes for identified risk factors were pooled as odds ratios (ORs) with 95% confidence intervals (CI). Heterogeneity was assessed using the I² statistic, with thresholds for low (<25%), moderate (25–70%), and high (>70%) heterogeneity. Potential publication bias was evaluated using funnel plots for asymmetry. P<0.05 was considered significant.

## RESULTS

**Fig.1:**
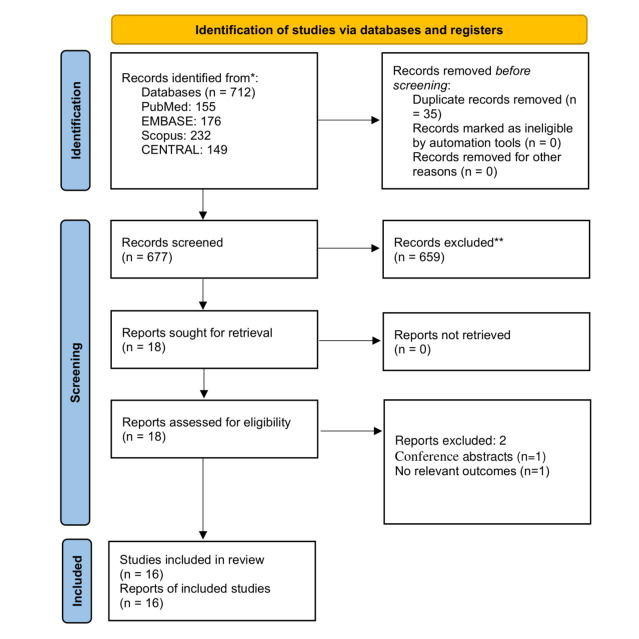
Study Selection Flow Chart

As detailed in the PRISMA flow chart (Fig.1), 712 records were identified from four databases (PubMed: 155, EMBASE: 176, Scopus: 232, CENTRAL: 149). After removing 35 duplicates, 677 records were screened based on titles and abstracts, excluding 659 irrelevant studies. Eighteen full-text reports were retrieved and assessed for eligibility, with two excluded (one conference abstract and one lacking relevant outcomes). Ultimately, 16 studies^10^-^25^ were included in the systematic review and meta-analysis.

The included studies were conducted across eight countries, with majority of studies originating from the United States,^12^,^15^,^18^–^20^,^23^,^24^ followed by China,^10^,^11^,^17^,^18^ Japan^16^,^25^ and one study each from Iran,^13^ Mexico,^14^ Turkey,^22^ and Finland.^21^

Regarding study design, most (n=12) studies^15^-^18^,^20^-^22^,^24^-^27^-^30^ were retrospective cohort, followed by two cross-sectional studies,^14^,^23^ one prospective cohort studies,^18^ and one randomized controlled trial.^13^

The total sample size across all studies was 165,589 participants, with individual study sizes ranging from 47^14^ to 66,001.^12^ The mean sample size was approximately 10,349 participants. The age of participants varied widely, with the mean or median age ranging from 46 years^16^ to 67.5 years.^21^ The overall mean age, where reported, was 55.1 years, representing a middle-aged to older population typically undergoing hysterectomy.

The studies reported a variety of surgical approaches. Abdominal hysterectomy was the most frequently performed procedure,^12^,^15^,^19^ followed by laparoscopic hysterectomy^10^,^16^ and vaginal hysterectomy.^18^,^23^ Several studies compared multiple surgical methods, such as the study by Guo et al. 2020,^18^ which assessed open, vaginal, laparoscopic, and robotic hysterectomies. Indications for surgery varied, focusing on malignant conditions,^17^,^24^ benign conditions,^18^,^19^ and studies encompassing both.^11^

The prevalence of SSIs varied significantly, ranging from 0.4% in the post-bundle group^18^ to 14.47%.^17^ The mean SSI prevalence across studies was approximately 6.8%. Factors influencing these rates likely include variations in patient populations, surgical techniques, and infection prevention protocols. Consistently reported risk factors included obesity or BMI >30 kg/m^2^, diabetes mellitus and prolonged operative time. Other factors, such as ASA score, smoking, postoperative serum albumin levels, and surgical approach, were also significant. Unique variables, like postoperative antibiotic duration^11^ and the use of Seprafilm,^21^ were highlighted in specific studies.

Follow-up durations were inconsistently reported. In studies that provided this information, follow-up durations averaged approximately 1.5 months. One study^17^ reported a shorter follow-up of 30 days. Table-II summarizes the characteristics of the included studies.

**Table-II:** General Characteristics of included studies.

Study Author and year	Study Duration and Year	Country	Study Design	Sample Size	Age	Type of Surgery	Type of Cancer	Operation Site	SSIs	Prevalence / Incidence rate (%) of infection	Risk Factors	Follow-up
Shao et al. 2024	January 2018 - December 2023	China	Retrospective cohort study	635	NR	Trans-hysterectomy	Benign and malignant indications	Abdomen and pelvis	Yes	8.5	BMI, hypo-proteinemia, postoperative antibiotic duration ≥ 3 days, history of prior abdominal surgery, total hospital stay ≥ 10 days, and malignancy pathology	NR
Xing et al. 2024	2019-2024	China	Single-center retrospective cohort study	301	NR	Laparoscopic hysterectomy	Cervical cancer	NR	Yes	12.60%	Age ≥ 60, BMI ≥ 24.0, diabetes, systemic immune-inflammation index (SII) ≥ 830, albumin/fibrinogen ratio (AFR) < 9.25, and neutrophil/ lymphocyte ratio (NLR) ≥ 3.45	1 month
Sabet et al. 2023	2020	Iran	Randomized double-blind controlled clinical trial	104	50.9 ± 9.3 years	Elective abdominal hysterectomy	NR	Abdomen	Yes	5.80%	Frequencies of infection among patients with a bleeding volume of more than 500 mL, diabetes, BMI > 30 kg/m2, and patients with higher hospitalization duration	NR
Tserenpuntsag et al. 2023	January 2015-December 2018	USA	Retrospective cohort study	66,001 ( SSI= 1,093)	52 ± 12 years	Abdominal hysterectomy	Gynecological cancer	Abdomen	Yes	1.66%	Age, obesity, open approach, wound class, ASA score, emergency at the time of procedure, procedure duration, diabetes, gynecological cancers, and transfusion	NR
De Mari et al. 2022	January 2015 - December 2019	USA	Retrospective cohort study	739	56.0 ± 12.3 years	Total abdominal hysterectomy	Gynecological cancer	Abdomen	Yes	Pre-protocol group= 5.9% ; abdominal closure protocol= 8.1%	Class 3 obesity (BMI >40), diabetes, Bowel resection, ASA class 3 or 4, Closing tray protocol	NR
Shi et al. 2021	Sept 2018-August 2020	China	Retrospective cohort study	318	NR	Laparoscopic hysterectomy, open hysterectomy	Endometrial carcinoma	Abdomen	Yes	14.47%	FIGO stage IV, open surgery, durations of drainage ≥ 7 d, postoperative serum albumin < 30 g/L, postoperative blood sugar ≥ 10 mmol/L	NR
Tsuzuki et al. 2021	2014-2018	Japan	Retrospective cohort study	1559	46.0 (43.0–49.0) Years	Laparoscopic hysterectomy	Benign gynecological cancer	Vagina	Yes	4.60%	Demographic and clinical variables, and patient- and surgery-related factors, current smoking, use of Seprafilm, CRP level on POD2, and postoperative vaginal cuff hematoma	NR
Carranza-Lira et al. 2020	NR	Mexico	Prospective, cross-sectional, study	47	47 (40-74) Years	Total abdominal (open) hysterectomy	NR	Abdomen	Yes	NR	HbA1c level, age, BMI, panniculus adiposus thickness, surgical time, trans-surgical bleeding, use of antibiotic prophylaxis, pre operative antisepsis	NR
Guo et al. 2020	2015 - 2018	USA	Prospective cohort study	Pre bundle= 515, post- bundle= 532 (total=1047)	Pre bundle= 50.0 ± 10.7 Years, Post bundle= 52.4 ± 11.4 Years	open, vaginal, laparoscopic, or robotic hysterectomy	Benign and malignant indications	Abdomen, Vagina	Yes	Pre bundle= 2.7%, post- bundle= 0.4%	Age, BMI, diabetes, race, type of surgery, type of surgeon	NR
Arakaki et al. 2019	2012 - 2014	Japan	Retrospective cohort study	107	55 (24 - 83) Years	Total abdominal hysterectomy, radical hysterectomy	Endometrial carcinoma	Abdomen	Yes	9.30%	Age, BMI, diabetes mellitus, current smoker,hypertension, Respiratory disease, Neurological risk, Operative time, Total blood loss (ml)	NR
Brown et al. 2019	2012 - 2014	USA	Retrospective cohort study	46 755	47 ± 11 Years	Laparoscopic Hysterectomy	Benign indication	Abdomen, Vagina	Yes	0.80%	Type of hysterectomy, age, race, smoking, preoperative steroid use, dialysis, ASA class, uterine weight, operative time, perioperative transfusion, hospital length of stay, wound classification, and performance of concurrent sacrocolpopexy	NR
Inci et al. 2016	January 2015- July 2015	Turkey	Retrospective study	103	62 Years	Endometrial cancer surgery	Endometrial carcinoma	NR	Yes	11.65%	Age, BMI, diabetes mellitus, ASA Score>2,cigarate smoking,hypertension, drain, Operation time, Total blood loss (ml), Pre-operative Hb (g/dl), Pre-operative glucose (mg/dl)	NR
Tuomi et al. 2016	2007 - 2013	Finland	Retrospective cohort study	1164	67.5± 10.5 Years	Open, Laparoscopic Hysterectomy	Endometrial carcinoma	Abdomen, vagina and pelvis	Yes	8.10%	Obesity, diabetes, and long operative time, smoking, conversion to laparotomy, and lymphadenectomy	NR
Uppal et al. 2016	July 2012 - February 2015	USA	Retrospective cohort study	21,358	48.1 ± 11.7 Years	Abdominal, vaginal, laparoscopic, or robotic hysterectomy	Gynecologic malignancy for benign indications	Abdomen, vagina and pelvis	Yes	2.06%	Preoperative prophylactic antibiotic, Surgical time, BMI, smoking status, ASA category, Surgical complexity, diabetes, Final pathology, Surgical route	NR
Bakkum-Gamez et al. 2013	January 1999 - December 2008	USA	Retrospective Cohort study	1369	64.2 ± 11.6 Years	Laparotomy, laparoscopy or robotic, vaginal hysterectomy	Endometrial carcinoma for benign indications	Abdomen, vagina	Yes	9.90%	BMI, ASA score >2, diabetes mellitus, pulmonary dysfunction such as sleep apnea or chronic pulmonary obstructive disease, and preoperative anemia (hematocrit <36%)	NR
Lake et al. 2013	2005 - 2009	USA	Cross-sectional Study	13,822	NR	(ASCH), (TVH),(LAVH), (TLH), and (LASCH)	NR	Abdomen, vagina and pelvis	Yes	2.70%	Hysterectomy route, operative time, ASA class 3 or greater, diabetes mellitus, and obesity category (BMI ≥40 kg/m2)	30-day postoperative follow-up

BMI- Body Mass Index; ASA- American Society of Anaesthesiologists; CRP – C-Reactive Protein; NR- Not reported; Hb- Hemoglobin; USA- United States of America; LASCH- Laparoscopic supracervical hysterectomy; TLH - Total laparoscopic hysterectomy; LAVH - Laparoscopic-assisted vaginal hysterectomy; TVH - Total vaginal hysterectomy; ASCH - Abdominal supracervical hysterectomy; FIGO - International Federation of Gynecology and Obstetrics;

### Meta-analysis:

**Fig.2:**
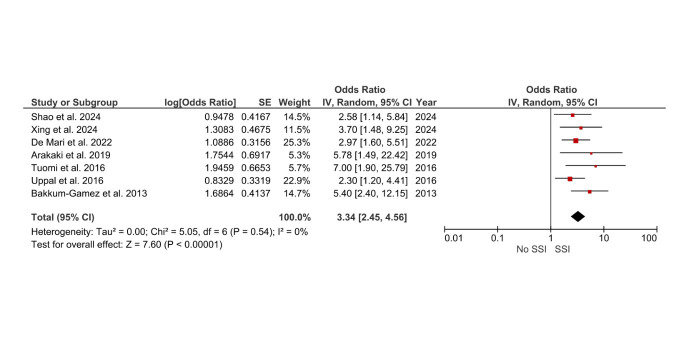
Forest plot: Effect of BMI >30kg/m2 on surgical site infection risk after hysterectomy.

The meta-analysis assessed various risk factors for SSIs after hysterectomy, integrating data from multiple studies to provide pooled impact estimates. Obesity (BMI >30 kg/m²) significantly increased the risk of SSIs, with a pooled OR of 3.34 (95% CI: 2.45–4.56), highlighting the role of impaired wound healing and reduced vascularity in obese patients (Fig.2).

**Fig.3:**
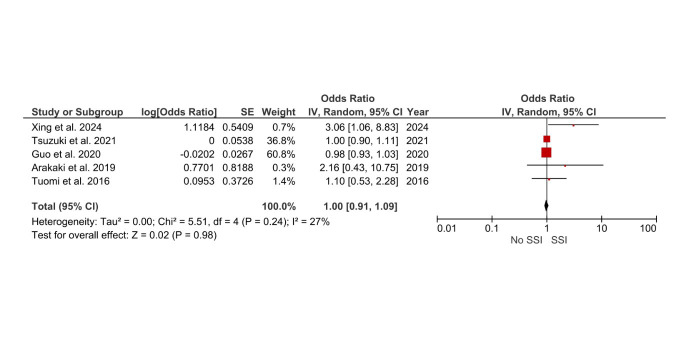
Forest plot: Effect of Age on surgical site infection risk after hysterectomy.

Age as a risk factor, with older patients exhibiting similar susceptibility to SSIs (OR = 1.00, 95% CI: 0.91–1.09), p=0.98, showed no statistical significance (Fig.3). Similarly, diabetes mellitus emerged as a strong predictor of SSIs, with an OR of 1.82 (95% CI: 1.41–2.35), with a p-value of <0.00001. Smoking, a modifiable risk factor, was associated with a 1.76-fold increased risk of SSIs (OR = 1.76, 95% CI: 1.37–2.26) with a p-value of <0.00001. Prolonged operative time (>2 hours) was linked to a nearly 1.5 -fold increased likelihood of SSIs (OR = 1.59, 95% CI: 1.19–2.11), p=0.002, underscoring the importance of efficient surgical workflows to minimize tissue exposure and microbial contamination.

Patients with (ASA) scores >3, reflecting higher baseline comorbidities, were at a significantly increased risk of SSIs (OR = 1.81, 95% CI: 1.51– 2.17) with a p-value of <0.00001. Similarly, blood transfusions were associated with an OR of 1.13 (95% CI: 0.91–1.41), p=0.27, indicating no potential link between transfusion-related immunomodulation and infection susceptibility.

Additional risk factors included prolonged hospital stays (>1 day) (OR = 2.47, 95% CI: 1.78–3.43, I² = 68%), duration of drainage >7 days (OR = 1.85, 95% CI: 0.95–3.60, I² = 60%), and infected wounds (OR = 2.62, 95% CI: 1.87–3.66, I² = 89%).

Patients undergoing open approach hysterectomy had nearly a threefold increased risk of SSIs (OR = 2.88, 95% CI: 2.24–3.72, I² = 35%) compared to minimally invasive approaches. Similarly, bowel resection, often performed in complex oncologic surgeries, was associated with the highest risk among all factors (OR = 3.07, 95% CI: 1.71–5.49, I² = 57%). Only one randomized clinical trial^18^ with low risk of bias was included in this review. And rest of the observational studies were of good quality with NOS score ranging between 6-8. (Table-III) No publication bias was detected and confirmed using visual inspection of plots.

**Table-III:** Quality Assessment of Observational studies using Newcastle-Ottawa Scale.

Study	Selection				Comparability		Outcome		Total
	Representativeness of the exposed cohort	Selection of the nonexposed cohort	Ascertainment of exposure	Demonstration that outcome of interest	Basis of the design or analysis	Assessment of outcome	Follow-up long enough for outcomes	Adequate follow up	
Shao et al. 2024	0	1	1	1	1	1	1	1	7
Xing et al. 2024	0	1	1	1	1	1	1	1	7
Tserenpuntsag et al. 2023	1	1	0	1	1	1	1	1	7
De Mari et al. 2022	1	1	1	1	1	1	1	1	8
Shi et al. 2021	1	1	0	1	1	1	1	1	7
Tsuzuki et al. 2021	1	1	1	1	1	1	1	1	8
Carranza-Lira et al. 2020	1	1	1	1	1	1	1	1	8
Guo et al. 2020	1	1	1	1	1	1	1	1	8
Arakaki et al. 2019	0	1	1	1	1	1	1	1	7
Brown et al. 2019	1	1	0	0	1	1	1	1	6
Inci et al. 2016	1	1	0	1	1	1	1	1	7
Tuomi et al. 2016	1	1	0	1	1	1	1	1	7
Uppal et al. 2016	0	1	1	1	1	1	1	1	7
Bakkum-Gamez et al. 2013	1	1	1	1	1	1	1	1	8
Lake et al. 2013	1	1	0	1	1	1	1	1	7

## DISCUSSION

This systematic review and meta-analysis aimed to evaluate the risk factors associated with SSI following hysterectomy. The included studies spanned diverse geographic regions and varied in design, providing a comprehensive understanding of factors contributing to SSIs post-hysterectomy. An overall mean SSI prevalence in the entire cohort was 6.8%, with rates ranging from 0.4% to 14.47%. Common risk factors for SSIs included obesity, diabetes mellitus, prolonged operative time, smoking, and higher ASA scores > 3. These findings emphasize the multifactorial nature of SSIs, with both patient-related and procedural factors playing significant roles. The results also underscore the importance of infection prevention strategies tailored to high-risk patients.

Hysterectomy remains one of the most commonly performed gynecological surgeries worldwide.^26^,^27^ Indications for the procedure include both benign conditions, such as uterine fibroids and abnormal uterine bleeding, and malignant conditions, such as endometrial and cervical cancers.^28^ Several included studies focused on cervical cancer, highlighting its surgical complexity and associated risks.^10^,^12^,^15^,^16^,^18^,^20^ Cervical cancer, a leading cause of cancer-related mortality among women, often requires radical hysterectomy, which is associated with higher morbidity, including an increased risk of SSIs.^29^

SSIs following hysterectomy significantly impact patient recovery, increasing hospital stays, readmission rates, and healthcare costs. Secondary infections often result from wound contamination during surgery, impaired immune responses, or prolonged operative time.^30^–^32^ This study showed that the incidence of SSIs varied depending on the surgical approach (abdominal, laparoscopic, or vaginal) and the comorbidities, which is consistent with previous research. Abdominal hysterectomy, for instance, is often associated with higher SSI rates compared to laparoscopic or robotic approaches due to the larger incision size and greater exposure of surgical sites.^33^

The analysis revealed that obesity increases the risk of SSIs by more than two-fold. This association aligns with previous studies^8^,^34^ highlighting how increased adiposity impairs vascularity, delays wound healing, and creates an environment conducive to bacterial growth.^35^ Similarly, diabetes mellitus significantly elevates the risk of infections due to hyperglycemia-induced immune suppression and delayed tissue repair.^36^ These findings underscore the importance of preoperative optimization for obese and diabetic patients to reduce SSI risk.

This study showed that older patients did not show any significant risk to SSIs. Advanced age is often associated with declining immune function and a slower healing process, particularly in patients undergoing complex procedures such as hysterectomy for malignancies.^37^ The impact of smoking was equally pronounced, increasing the odds of infection by 1.83 times. Smoking impairs tissue oxygenation and vascularity, exacerbating surgical site complications,^38^ making smoking cessation an essential component of preoperative care.

The study demonstrated a significant association of procedure-related factors with the incidence of SSIs. Extensive operative time (>2 hours) was associated with nearly a threefold increased risk of SSIs, likely due to a prolonged tissue exposure and microbial contamination.^30^ Compared to minimally invasive methods, open surgical approaches was linked to a significantly higher SSIs risk, reinforcing the advantages of laparoscopic and robotic techniques in reducing infection rates. Similarly, blood transfusions and ASA scores >3 highlighted the interplay between procedural complexity, patient comorbidities, and immune responses in increasing infection susceptibility.^39^ Specific conditions, such as prolonged hospital stays (>1 day), duration of drainage >7 days, and infection, further compounded the risk of SSIs. The association of bowel resection with the three-fold odds ratio among all factors highlights the complexity and infection-prone nature of extensive surgical interventions, often performed in cases of advanced cervical cancers.

In the context of cervical cancer, these risk factors become particularly relevant, as surgeries for malignant conditions, such as radical hysterectomy, are inherently more complex, often require extensive tissue dissection, lymphadenectomy, or concurrent procedures, involve longer operative times and larger surgical fields further increasing the risk of SSIs compared to procedures for benign indications.^40^,^41^ Moreover, patients with cervical cancer may present with immunosuppression due to advanced disease stages or prior chemoradiotherapy, compounding the susceptibility to infections. For instance, patients with advanced disease, such as those in FIGO stage IV were found to be at a higher risk of SSIs due to prolonged operative times, impaired wound healing, and systemic immune dysfunction.^17^ Anastomotic leaks, also particularly in the context of gynecologic oncology procedures involving bowel resection, have been strongly associated with adverse postoperative outcomes, including a significantly increased risk of surgical site infections.^42^

Despite these findings, the review identified considerable heterogeneity among the included studies. It is possible that this variability stems from the variations in patient populations, surgical techniques, SSI definitions, and follow-up durations. While some studies exclusively focused on malignant indications, such as endometrial or cervical cancer, others included mixed populations with benign conditions, introducing variability in infection rates. Differences in healthcare resources, surgical protocols, and antibiotic prophylaxis further contributed to the observed variability, affecting the generalizability of findings. For example, studies from high-resource settings like the USA often incorporated advanced infection prevention methods^18^ that lowered SSI rates, whereas resource-limited settings may face challenges in implementing such protocols.

### Limitations:

This review has several strengths and limitations. The strengths include the systematic synthesis of data from various studies conducted across multiple countries, ensuring a comprehensive and diverse analysis. The inclusion of various study designs and surgical approaches enhanced the generalizability of the findings, while the use of rigorous inclusion and exclusion criteria minimized bias, improving reliability. However, significant heterogeneity among studies limited the ability to pool data in certain analyses. Variations in SSI definitions and follow-up durations may have resulted in underreporting or inconsistencies, and some studies lacked detailed reporting of confounding variables, which could influence the observed associations between risk factors and SSIs. A notable limitation of this review is the geographic concentration of included studies, with nearly half originating from the United States. A major limitation is the pooling of benign, oncologic, and mixed hysterectomy cases without subgroup analysis. These populations differ markedly in surgical complexity and baseline risk, particularly with oncologic procedures having higher complication rates. This heterogeneity may introduce confounding and limits the validity and clinical interpretability of the pooled results.

This may introduce bias related to healthcare system differences, surgical protocols, and resource availability, potentially limiting the generalizability of findings to low- and middle-income settings. Only a minority of the included studies explicitly reported infection prevention bundles or detailed antibiotic protocols. Where described, these strategies included perioperative SSI prevention bundles^18^, closure-focused protocols such as dedicated closing tray or abdominal closure protocols^15^, optimization of prophylactic antibiotic selection in line with guidelines^20^, adjunctive topical antimicrobial use such as preoperative vaginal metronidazole gel in addition to standard systemic prophylaxis^13^, and postoperative antibiotic duration or perioperative antisepsis practices.^11^,^14^ Collectively, these reports highlight substantial variability and frequent underreporting of infection prevention practices across studies. Additionally, follow-up duration was inconsistently reported, with most studies lacking clear timelines for postoperative assessment. This variability may affect the detection and reporting of SSIs, introducing potential detection bias that could influence the observed associations. The inconsistency in threshold definitions for key risk factors across studies could possibly contribute to the heterogeneity. For instance, BMI cutoffs and operative time thresholds varied notably, with some studies of united studies using a BMI >30 kg/m² considered for obesity and Asian studies considering BMI >25 kg/m^2^ for the same. Despite these limitations, the review provides valuable insights into modifiable and non-modifiable risk factors for SSIs, emphasizing the importance of targeted interventions to improve patient outcomes.

## CONCLUSION

This systematic review and meta-analysis identified key risk factors for SSIs following hysterectomy, including obesity (BMI > 30 kg/m²), diabetes, smoking, prolonged operative time (>2 hours), and higher ASA scores (ASA score > 3). The findings also highlighted procedure-related factors such as open surgical approaches, blood transfusions, prolonged hospital stays, and bowel resection, which were strongly associated with increased SSI risk. These findings have direct clinical implications, suggesting that existing SSI risk scores should be updated to reflect high-impact factors such as obesity, diabetes, prolonged operative time, and ASA scores. Incorporating these into preoperative risk calculators could improve SSI prediction and guide targeted interventions. Clinicians should prioritize modifiable risk factors through optimized patient preparation and consider minimally invasive approaches where feasible to reduce infection risk following hysterectomy. By implementing tailored prevention protocols and further exploring advanced surgical technologies, the burden of SSIs post-hysterectomy can be significantly reduced, leading to improved patient outcomes and substantial reductions in healthcare costs.

### Future recommendation:

Future research should focus on standardizing SSI definitions and follow-up durations to facilitate comparability across studies. Prospective studies with more extensive, multicenter cohorts are needed to validate the findings and assess the effectiveness of targeted interventions. Additionally, exploring the impact of advanced surgical technologies, such as robotic-assisted hysterectomy, on SSI rates could provide valuable insights. Finally, implementing evidence-based infection prevention measures in clinical practice and evaluating their outcomes should be prioritized.

### Authors’ contributions:

**YY and TZ:** Study design, literature search and manuscript writing.

**YY and TZ:** data collection, data analysis and interpretation. Critical review.

**TZ:** manuscript revision and validation and is responsible for the integrity of the study.

All authors have read and approved the final manuscript.
